# Report of Committee on Army Legislation

**Published:** 1894-11

**Authors:** F. L. Kilborne

**Affiliations:** Chairman; Philadelphia, Pa.


### Report of Committee on Army Legislation, Thirty-first Annual Meeting United States Veterinary Medical Association.

Philadelphia, Pa., Sept., 1894.

Efforts to procure satisfactory legislation during the past session of Congress, for the reorganization and improvement of the

United States Army Veterinary Service, have met with the same failure that has attended the endeavors of previous years.

Several bills, five or six, I believe, were introduced early in the session, but none of them passed beyond the Committee rooms.

The bill which seemed to meet with the most general favor with the Army veterinarians was introduced in the Senate by Senator Pettigrew, and in the House by Representative Pickler. This bill is almost a counterpart of that introduced at the last session by Senator Cameron and Representative Robinson, of Pennsylvania, and is given in full below. I regret that I have not the copies of the other bills with me.

The bill was favorably received by War authorities, and, with two or three unimportant changes in the phraseology, received their unqualified endorsement.

With the Military Committees in Congress, our great obstacle was the fact that several bills were introduced, instead of uniting upon and introducing a single bill. The members informed us that until the veterinarians could unite upon some one measure, they could expect but little encouragement from the Committees.

On behalf of the Army veterinarians, Dr. John Tempany, 9th Cavalry, was sent to Washington in the interest of Army veterinary legislation, but returned early to this post without having accomplished anything.

If any Army veterinary legislation is to be secured, we believe that it will be necessary for the veterinarians to first unite and agree upon some definite policy. Then to have a single bill drawn and introduced, instead of the several private bills.

We are informed that even among the Army veterinarians there are those who have been working as earnestly against the proposed legislation as the friends of the measure were in working for it. Concerted action should first be secured, if we are to hope for success.

We also believe that a small appropriation should be voted by the Association for the use of the next Committee on Army Legislation, to enable it to carry on its work more satisfactorily.

F. L. KILBORNE, *Chairman.*

